# A deep ensemble model to predict miRNA-disease association

**DOI:** 10.1038/s41598-017-15235-6

**Published:** 2017-11-03

**Authors:** Laiyi Fu, Qinke Peng

**Affiliations:** Systems Engineering Institute, School of Electronic and Information Engineering, Xi’an Jiaotong University, Xi’an, Shannxi 710049 China

## Abstract

Cumulative evidence from biological experiments has confirmed that microRNAs (miRNAs) are related to many types of human diseases through different biological processes. It is anticipated that precise miRNA-disease association prediction could not only help infer potential disease-related miRNA but also boost human diagnosis and disease prevention. Considering the limitations of previous computational models, a more effective computational model needs to be implemented to predict miRNA-disease associations. In this work, we first constructed a human miRNA-miRNA similarity network utilizing miRNA-miRNA functional similarity data and heterogeneous miRNA Gaussian interaction profile kernel similarities based on the assumption that similar miRNAs with similar functions tend to be associated with similar diseases, and vice versa. Then, we constructed disease-disease similarity using disease semantic information and heterogeneous disease-related interaction data. We proposed a deep ensemble model called DeepMDA that extracts high-level features from similarity information using stacked autoencoders and then predicts miRNA-disease associations by adopting a 3-layer neural network. In addition to five-fold cross-validation, we also proposed another cross-validation method to evaluate the performance of the model. The results show that the proposed model is superior to previous methods with high robustness.

## Introduction

MiRNAs are a special type of short endogenous non-coding RNA with a length of ~22 nt. MiRNAs are usually regarded as gene repressors at the post-transcriptional level through binding to the 3′-UTRs of the target mRNAs^1–4^. Nevertheless, miRNAs show a positive influence in regulating genes according to some studies^5^. Enormous numbers of miRNAs have previously been discovered, including 2588 human genome miRNAs reported in miRBase^6^. Substantial evidence indicates that miRNAs play a vital role in regulating biological processes such as cell development^7^, proliferation^8^, differentiation^9^, etc. In particular, miRNA dysregulation is related to many human diseases through many factors, including, for example, miRNA-mRNA interactions^10^, miRNA-lncRNA (long non-coding RNA) interactions^11,12^, miRNA-protein interactions^13^, miRNA-environmental factors interactions^14^, and so on. Among these miRNAs, miR-15 and miR-16 are the first two miRNAs reported to interact with cancers. Calin *et al*. clarified that miRNAs are deleted in over half of B-cell chronic lymphocytic leukaemia (B-CLL)^15^ instances. MiR-129, miR-142-5p and miR-25 showed differentially expressed phenomenon in all paediatric brain tumour types^16^. Accumulated evidence also revealed that the regulation of Ad6 by miR-122 could be significantly helpful in improving the anticancer efficacy of prostate cancer^17^. Therefore, the inferring associations between miRNAs and diseases could effectively boost the detection of disease biomarkers for cancer treatment, diagnosis, and furthermore, prevention.

By collecting data from biological experiments and raising evidence of connections between miRNAs and diseases, two databases called HMDD^18^ and miR2Disease^19^ were constructed. These databases may provide a comprehensive resource for miRNA deregulation in various human diseases. Due to the fast development of computational intelligence, various types of powerful computational methods have emerged with the goal of predicting miRNA-disease associations^14,20^. Among these methods, many require similarity computation for miRNAs and diseases to accomplish association prediction. Zou *et al*.^21^ reviewed the main similarity computational methods. By integrating different source datasets, many approaches have been developed to compute the similarity matrices and give a final prediction regarding the miRNA-disease association. Both machine learning based methods and network-based methods are frequently used in these approaches.

The machine-learning-based methods mainly focus on improving classification accuracy and prediction performance using features extracted from raw data^22,23^. Many studies have proposed approaches that involve machine-learning algorithms. A typical support vector machine based method was used to predict each miRNA-disease relation^24^. The lasso regression method was proposed using protein related diseases and miRNAs connections to infer miRNA-disease associations^25^. Furthermore, Jiang *et al*. adopted a Naive Bayes model to prioritize disease-related miRNAs using genomic data^26^. However, these methods require both positive and negative samples to train the models; therefore, a semi-supervised method using regularized least squares (RLS) was proposed to make predictions without using negative samples^27^. A similar method called Laplacian Regularized Least Squares (LRLS) was adopted to predict long non-coding RNAs and disease association prediction^28^. This method achieved good success. Another machine-learning based method was RBMMMDA, which utilized a restricted Boltzmann machine (RBM) to predict miRNA-disease association types; however, this method required lots of parameters^29^.

Network approaches to predict diseases have been successfully applied to explore the relationship between diseases and genes^30^. Observing the influence and causes of diseases such as cancers via networks has become popular. Network-based methods are typically based on the common assumption that functional related miRNAs tend to be associated with phenotypically similar diseases, and vice versa^31,32^. Jiang *et al*. proposed a computational model based on hyper-geometric distribution to detect human miRNA-disease associations^33^. However, this method relied heavily on predicted association, which results in high false-positive and false-negative rates. Considering that many miRNAs can regulate target genes that may finally cause different diseases if not properly expressed, those gene-related diseases could also have potential associations with gene-related miRNAs. Based on that, Shi *et al*. presented a random walk algorithm by integrating a protein-protein interaction (PPI) network and utilizing functional links between disease genes and miRNA targets genes^34^. Furthermore, protein related diseases and miRNAs could also contribute to the inference of novel miRNA-disease associations^35^. However, the methods mentioned above were restricted by the incomplete disease-related gene/protein network and result in low true-positive rates. Thus, using more data is necessary. Consequently, Wang *et al*. proposed a method to calculate miRNA functional similarity (MISIM) using miRNA-disease associations and a directed acyclic graph (DAG) of disease annotation. Chen *et al*. discussed a model called WBSMDA that combined miRNA functional similarity, disease semantic similarity, and Gaussian interaction profile kernel similarity to obtain a more reliable prediction result^36^. Chen *et al*. also presented a network similarity-based model using a random walk with restart algorithm (RWRMDA) to predict miRNA-disease associations^37^. Similarly, Chen further proposed an improved random walk with restart algorithm called HGIMDA to boost the performance of the traditional RWRMDA^38^. Recently, two methods that used k-nearest-neighbour (KNN) methods, SDMMDA^39^ and RKNNMDA^40^, were proposed and gained satisfactory results. You *et al*. proposed a path-based method and adopted depth-first algorithms to infer potential miRNA-disease associations^41^ and further improve the performance. Furthermore, Li *et al*. applied a low-rank matrix recovery method to uncover missing miRNA-disease associations^42^, and Chen *et al*. proposed a model called DRMDA^43^ that utilized a sparse auto-encoder to obtain the representation of miRNA and disease and then chose a SVM classifier to predict miRNA-disease associations.

Previous computational models were limited to low true-positive rates because of the lack of non-linear relationship capture. Deep learning is a recently developed approach that provides applicable solutions, especially for large datasets and non-linear pattern analyses^44,45^. Deep learning models often play a role of hybrid multiple-layer abstractors by mapping the data to a high-level feature space where the prediction model can be constructed. These approaches have been successfully applied in mutiple bioinformatics scenarios, such as gene expression^46^, DNA-protein binding prediction^47^, etc. Deep learning models have been shown to greatly enhance the performance and they show satisfactory results. Due to their powerful ability to capture hidden, complicated, non-linear connections from original data, deep learning models can even act as feature extractors to supplement the original feature input^48^. The number of miRNA/disease samples is quite small compared to other biological problems; however, the total number of miRNA-disease pair-wise connections is quite large. Therefore, to implement the proposed model using deep learning, every pair-wise miRNA-disease connection is used as one sample to train the network, which helps overcome the lack of training samples.

Integrating more heterogeneous data is also helpful in enhancing the prediction performance^49^. Therefore, in this study, we integrated multiple datasets related to miRNAs or diseases and then developed a novel deep ensemble framework, DeepMDA, to predict miRNA-disease associations using deep learning. DeepMDA first utilizes a deep learning model to extract high-level features from miRNA and disease similarity matrices; then, a three-layer neural network classifier was implemented to make predictions between miRNA-disease pairs. DRMDA was also observed using an auto-encoder to extract features, but its structure, computational cost and robustness are different from DeepMDA; our proposed model gained better results compared to DRMDA. Overall, our model showed superior performance than did the other five classical miRNA-disease association prediction models in both 5-fold cross-validation and leave-one-disease-out cross-validation.

## Results

### Five-fold cross validation

Cross-validation is a frequently used method in machine learning and can greatly reduce the bias caused by sample selection. In this case, evaluating the performance of different models is crucial and practical when some of the positive miRNA-disease associations are missing or a new miRNA-disease association is added. To evaluate the prediction performance of DeepMDA, we adopted 5-fold cross-validation compared with other five state-of-the-art computational models (i.e., RLSMDA, HGIMDA, NCPMDA, PBMDA, RKNNMDA). RWRMDA was a representative approach in the domain and was often considered as a standard method to validate performance. HGIMDA was an improved version of RWRMDA that incorporated changes in the data pre-processing procedure. RLSMDA was a semi-supervised method often listed as a compared method in miRNA-disease studies. NCPMDA was a network consistency projection method that showed superior performance compared to HDMP^50^ and NetCBI^51^. PBMDA is a path-based method that adopted a depth-first search algorithm to infer potential miRNA-disease associations^41^. RKNNMDA is a KNN-based model that was combined with the SVM rank method to predict miRNA-disease associations^40^. Other recently developed methods such as MCMDA^52^ and ILRMR^42^, whose datasets used in their studies differ from ours; therefore, we did not choose them for comparison. In the 5-fold cross-validation, all the known interactions were randomly split into 5 subsets with equal size. In each fold, one subset was left out as testing samples, and the remaining four subsets were treated as training sets. The entire procedure was repeated until the entire subset was used for training. The average performance was adopted for evaluation.

The receiver-operating characteristics (ROC) curve was chosen by plotting the true positive rate (TPR, sensitivity) curve against the false positive rate (FPR, specificity) at different thresholds. Specificity is the proportion of samples below the given threshold and sensitivity represents the percentage of samples higher than the threshold. The area under the ROC curve (AUC) was also calculated to evaluate the ability of the prediction model. An AUC value of 1 denotes that the performance is perfect, and an AUC value of 0.5 indicates random prediction performance. Furthermore, we also adopted another type of quality measure used in these types of studies called AUPR (Area Under the Precision vs. Recall Curve). Due to the unbalanced phenomenon of the dataset, the positive data were smaller compared to the negative data. Therefore, AUPR was proposed to reduce the impact caused by a high proportion of false positive data. Similar to the AUC score, AUPR values closer to 1 indicate that the performance is better.

The 152 miRNAs in *SM*_*T*_ and 255 miRNAs in *SM*_*F*_ are separately utilized with 383 disease similarities to evaluate the performance in small datasets of miRNAs. The experiments implemented 5-fold cross-validation, and the results are shown in Tables 1 and 2. The results show that DeepMDA achieved the highest AUC and AUPR scores in both datasets compared to other algorithms. Furthermore, the three deep models that will be mentioned later had advantages compared to other network based algorithms.

**Table 1:** Results on the *SM*_*T*_ miRNA datasets.

Method	AUC	AUPR
RLSMDA	0.7715 ± 0.027	0.0321 ± 0.004
HGIMDA	0.7040 ± 0.025	0.0402 ± 0.006
NCPMDA	0.7760 ± 0.012	0.0578 ± 0.006
PBMDA	0.8194 ± 0.032	0.2106 ± 0.017
RKNNMDA	0.5678 ± 0.016	0.1581 ± 0.038
DeepMDA	0.9126* ± 0.004	0.2297* ± 0.040
SAE + ADA	0.8996 ± 0.010	0.1859 ± 0.026
RAW + DNN	0.9102 ± 0.007	0.1991 ± 0.028

The AUC and AUPR scores are listed above. The * indicates the highest AUC/AUPR score. Generally, the three deep learning models performed better than other five models.

**Table 2:** Results on the *SM*_*F*_ miRNA datasets.

Method	AUC	AUPR
RLSMDA	0.8325 ± 0.007	0.2457 ± 0.008
HGIMDA	0.7169 ± 0.005	0.1182 ± 0.004
NCPMDA	0.8849 ± 0.006	0.3473 ± 0.010
PBMDA	0.8925 ± 0.005	0.4867 ± 0.014
RKNNMDA	0.7044 ± 0.004	0.3365 ± 0.013
DeepMDA	0.9270* ± 0.005	0.5853* ± 0.031
SAE + ADA	0.8982 ± 0.005	0.4376 ± 0.024
RAW + DNN	0.9153 ± 0.004	0.5293 ± 0.026

The AUC and AUPR scores are listed above. The * indicates the highest AUC/AUPR score. Generally, the three deep learning models performed better than the other five models.

Next, we integrated multiple datasets and used all the 495 miRNAs in *SM* with the 383 disease similarities to evaluate the performances. The results of the five different approaches together with DeepMDA are shown in Table 3. DeepMDA, RLSMDA, HGIMDA, NCPMDA, PBMDA, RKNNMDA obtained average AUCs of 0.9486 ± 0.002, 0.8475 ± 0.005, 0.7689 ± 0.011, 0.8731 ± 0.007, 0.9086 ± 0.004, 0.7076 ± 0.005, respectively, in 5-fold cross-validation. DeepMDA also showed the highest AUPR score compared to the five previous methods in all three datasets.

**Table 3:** Results on the full miRNA-disease datasets.

Method	AUC	AUPR
RLSMDA	0.8475 ± 0.005	0.1157 ± 0.004
HGIMDA	0.7689 ± 0.011	0.1120 ± 0.007
NCPMDA	0.8731 ± 0.007	0.2801 ± 0.011
PBMDA	0.9086 ± 0.004	0.4378 ± 0.016
RKNNMDA	0.7076 ± 0.005	0.3534 ± 0.011
DeepMDA	0.9486* ± 0.002	0.5917* ± 0.014
SAE + ADA	0.9211 ± 0.002	0.4075 ± 0.011
RAW + DNN	0.9368 ± 0.001	0.4933 ± 0.014

The AUC and AUPR scores are listed above. The * indicates the highest AUC/AUPR score. Generally, the three deep learning models performed better than the other five models.

To validate the reasonability of proposed model, another two models were implemented based on the deep learning framework. In the first model, we constructed Stacked AutoEncoder(SAE) with Adaboost, called SAE-ADA, as one alternative classifier. Adaboost is an ensemble method often used in machine learning that obtains a more satisfactory result compared to other methods during experiments. In the second model, we used the latter part of DeepMDA, that is, the raw similarity data was used as input, removing the two stacked autoencoders and directly feeding the feature vector to a three-layer fully connected network to construct the classifier (RAW-DNN). We measured these two deep models (SAE + ADA, RAW + DNN) and calculated their AUC values for comparison with DeepMDA. As shown in Table 3, SAE + ADA achieved an average AUC score of 0.9211 ± 0.002 and RAW + DNN obtained an average AUC score of 0.9386 ± 0.001. Therefore, the two deep network models show promising results. We also compared the DRMDA model (i.e., single auto-encoder with SVM) and single auto-encoder with DNN model to validate their performances. As shown in Table 4, DeepMDA still achieved the highest AUC and AUPR scores.

**Table 4:** Comparison between DRMDA and DeepMDA on the full miRNA-disease datasets in five-fold cross validation.

Method	AUC	AUPR
Single SAE + SVM(DRMDA)	0.8812 ± 0.006	0.4614 ± 0.004
Single SAE + DNN	0.9394 ± 0.002	0.5003 ± 0.010
DeepMDA	0.9486* ± 0.002	0.5917* ± 0.014

The AUC and AUPR scores are listed above. Generally, DeepMDA performed better than the other two models.

**Figure 1:**
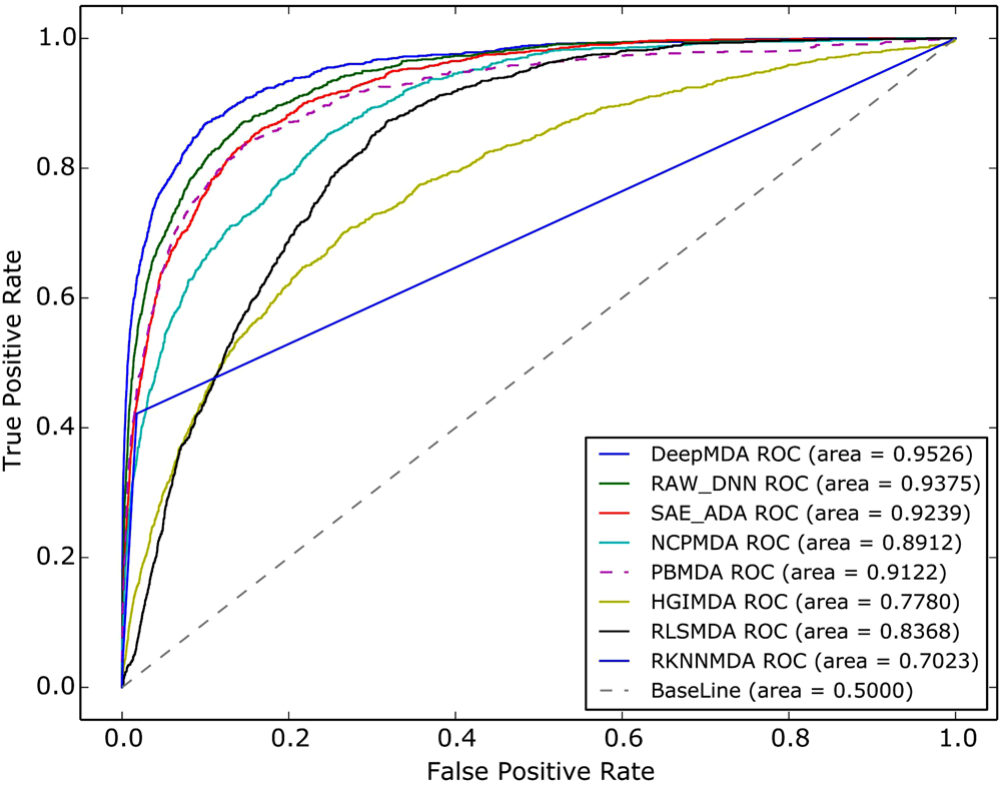
ROC curves and AUC values of eight different methods based on 5-fold cross-validation. The three deep learning models performed better than other 4 network-based methods in general. DeepMDA had the best performance in all the eight models.

From these results, it is clear that adopting the SAE as a high-level feature extractor is an essential aspect for improving performance when comparing DeepMDA with RAW + DNN. On the other hand, DNN still inevitably played the role of final classifier when we compared the result of the proposed model with SAE + ADA. Overall, DeepMDA showed a better result in ensemble deep network frameworks compared with two other deep models, and it achieved the best performance of all the compared methods. The standard error of each AUC was small in the five CVs’; therefore, we randomly chose one of the ROC results during 5-fold cross-validation, as depicted in Fig. 1.

### Leave-one-disease-out cross-validation

**Figure 2:**
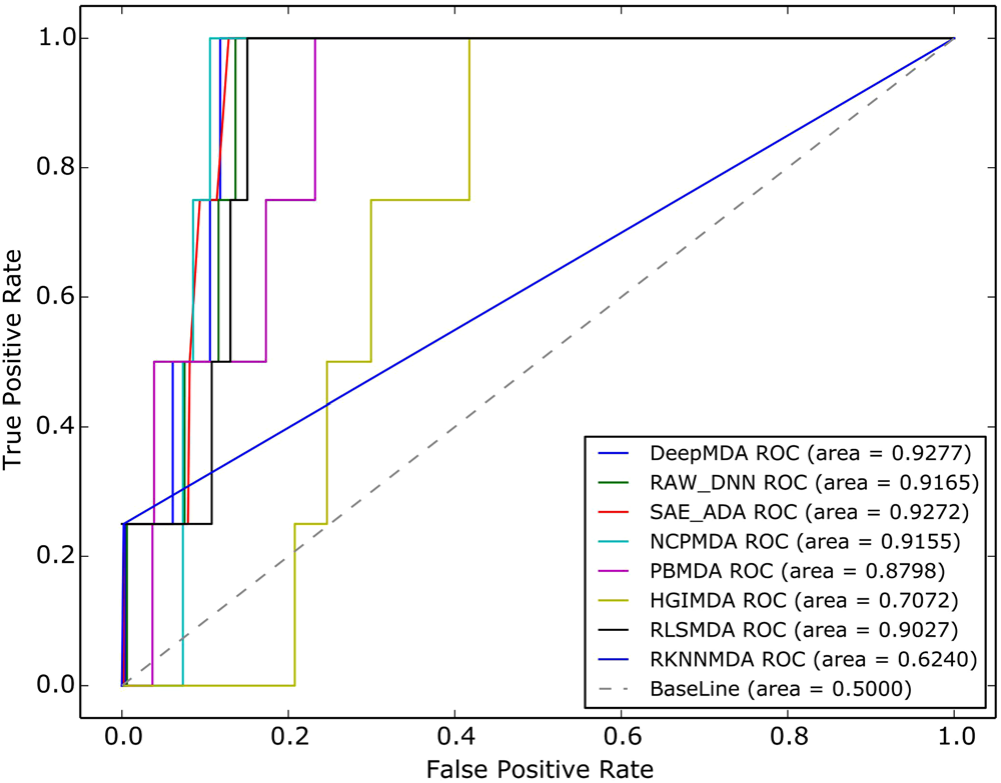
ROC curves and AUC values of eight different methods based on LODOCV. The three deep learning models performed better than other five network-based methods in general. DeepMDA had the best performance in all the eight models.

The traditional leave-one-out method of cross-validation (LOOCV) leaves one known miRNA-disease association out in each turn and uses other known associations for model training, and the method then uses that test sample ranking with all the other associations in every iteration. However, training samples were separated from test samples during every recursion in the proposed model, because it could induce bias if we used only one miRNA-disease association as the test sample. Thus, instead of leaving each known miRNA-disease association out and predicting it among all the unknown miRNA-disease association w.r.t. of the investigated disease in each turn, we left every column samples about one disease each time. This method was called Leave-One-Disease-Out Cross-Validation (LODOCV). In every iteration, we tried to predict all the chosen disease-associated miRNAs using the information of other disease-related miRNAs. To our knowledge, LODOCV is considerably more difficult to use than traditional LOOCV because we tried to uncover every miRNA-disease association w.r.t to each disease without any known miRNA-disease information. To be specific, we left all of the diseases as test samples in one iteration. Using other disease-related miRNA information to predict all the unknown disease-associated miRNA associations is a challenging and meaningful problem for researchers and medical diagnoses. Network-based models and three deep learning models were selected to evaluate the overall performance. The results are shown in Table 5. DeepMDA achieved an average AUC score of 0.8729, SAE + ADA obtained an average AUC score of 0.8552, and RAW + DNN reached an AUC score of 0.8633. Five network-based models (RLSMDA, NCPMDA, HGIMDA, PBMDA, RKNNMDA) achieved average AUC scores of 0.8530, 0.6374, 0.7616, 0.6902 and 0.5680, respectively. Regarding the AUPR scores, DeepMDA still achieved the highest AUPR score compared with the other methods. Likewise, we randomly picked one ROC result and drew the ROC curve as shown in Fig. 2. Overall, DeepMDA obtained the best performance in LODOCV compared with the other 7 methods.

**Table 5:** Results on the full miRNA-disease datasets in LODOCV.

Method	AUC	AUPR
RLSMDA	0.8530 ± 0.133	0.2066 ± 0.240
HGIMDA	0.7616 ± 0.164	0.1025 ± 0.142
NCPMDA	0.6374 ± 0.220	0.0596 ± 0.104
PBMDA	0.6902 ± 0.223	0.2918* ± 0.289
RKNNMDA	0.5680 ± 0.131	0.2085 ± 0.273
DeepMDA	0.8729* ± 0.118	0.2556 ± 0.271
SAE + ADA	0.8552 ± 0.124	0.1914 ± 0.220
RAW + DNN	0.8633 ± 0.121	0.2180 ± 0.248

The AUC and AUPR scores are listed above. Generally, DeepMDA performed better than the other seven models in LODOCV.

### Robustness in DeepMDA

The deep learning models showed powerful abilities in high-level feature extraction, especially in complex relationship analysis. To measure their abilities to capture the data structure and interaction relationship, we further implemented five-fold cross-validation using noisy data. We added some white noise data to the trained data obtained from the autoencoders, and then implemented a deep neural network classifier, an AdaBoost classifier and a random forest classifier separately to compare their performances. The latter two classifiers were chosen because they are both ensemble classifiers and they achieved more satisfactory prediction results during the experiments. The results in Table 6 show that the AUC score dropped from 0.9486 to 0.9334 using DeepMDA, but the AUC score dropped from 0.9211 to 0.8235 using SAE + ADA and from 0.9249 to 0.8122 using SAE + RF. The AUC score of DRMDA dropped from 0.8812 to 0.7757. This result illustrated that DeepMDA could capture the complex relationships and be robust when noise data were introduced.

**Table 6:** Results on the noisy miRNA-disease datasets.

Method	AUC	AUPR
Noised DeepMDA	0.9334 ± 0.005	0.4558 ± 0.019
Noised SAE + ADA	0.8235 ± 0.012	0.1613 ± 0.015
Noised SAE + RF	0.8122 ± 0.015	0.1320 ± 0.014
Noised DRMDA	0.7757 ± 0.044	0.1290 ± 0.034
DeepMDA	0.9486* ± 0.002	0.5917* ± 0.014
SAE + ADA	0.9211 ± 0.002	0.4075 ± 0.011
SAE + RF	0.9249 ± 0.003	0.5674 ± 0.012
DRMDA	0.8812 ± 0.006	0.4614 ± 0.004

The AUC and AUPR scores are listed above. Generally, DeepMDA performed better than other three models when adding noise.

### Case studies

We further investigated some complex human diseases to determine the disease-related miRNAs using the proposed model for measuring model prediction ability. The results showed that human digestive and urinary systems are occasionally deregulated through miRNA functional expression. The oesophagus and colon belong to the digestive system, while the kidneys belong to the urinary system. Therefore, we investigated the potential association between miRNAs and three different diseases, i.e., oesophageal neoplasms, kidney neoplasms and colon neoplasms. The prediction results were validated by checking the experimental results presented in two databases, miR2Disease^19^ and dbDEMC^53^, which record many experimentally verified miRNA-disease associations. We implemented LODOCV to predict candidate disease-related miRNAs for these three disease-related cases, and many miRNAs could be precisely predicted using DeepMDA. In total, 47, 42 and 44 out of the top 50 validated miRNAs were predicted w.r.t. colon neoplasms, oesophageal neoplasms, and kidney neoplasms, respectively (see details in Supplementary Table S1, Supplementary Table S2 and Supplementary Table S3).

Colon neoplasms are one of the most severe diseases worldwide^54^. It was reported that almost half of the patients with colon neoplasms die of metastatic disease within 5 years from diagnosis^55,56^. Increased evidence has indicated that miRNAs have potential associations with colon neoplasms. For instance, miR-145 may inhibit cell growth in colon neoplasms by targeting the insulin receptor substrate-1 ^57^. Furthermore, tumour specimens showed highly significant and large-fold change differential expression of the levels of several miRNAs, including miR-135b, miR-133a, miR-1, miR-31, and others^58^. MiR-20a and miR-155 were confirmed to be up-regulated in Colon Neoplasms^59^. By using DeepMDA, the potential colon neoplasm-related miRNAs were identified, and the results are listed in Supplementary Table S1, which shows that 10 out of the top 10 and 46 out of the top 50 predicted miRNAs were confirmed based on miR2Disease and dbDEMC. For example, an inverse correlation of miR-21 was found in 10 colorectal cell lines suggesting that it might play a role as a useful diagnostic biomarker for colon neoplasms prognosis^60,61^. To further validate the relationship between predicted miRNAs and cancers, various cancer hallmarks were verified, such as genes that are associated with miRNAs. For instance, some genes such as BRAF, APC, and TP53 can be regarded as colon cancer hallmarks^62^, and these gene-related miRNAs associations could be validated by miRTarBase^63^, showing that these miRNAs could possibly regulate these genes. We also found that many disease-related miRNAs are likely to be enriched together; this pattern is similar to disease-associated genes that play roles in some cancer hallmarks^30^, suggesting that these miRNAs may co-regulate some diseases such as cancers.

Oesophageal neoplasms are one of the most common malignant tumours worldwide and are ranked as the sixth main cause of cancer related deaths^64^. It has been reported that the overall 5-year survival rate is approximately 20% despite advanced treatments^56,65^. Improving the understanding of the biological mechanism underlying oesophageal cancer is crucial for diagnosis and disease prevention^66^. Experimental evidence has revealed that several human miRNAs are located at genomic regions related to the expression of tumour genes such as oesophageal neoplasms^67^. For instance, miR-155 and miR-103 are highly expressed in tumour tissues and could be correlated with different clinic pathologic classifications^68^. miR-98 may suppress migration and invasion in human oesophageal squamous cell carcinomas^69^. Using DeepMDA to predict potential oesophageal neoplasm-related miRNAs could help validate the prediction ability of our model. As a result, 8 out of the top 10 candidates and 42 out of the top 50 predicted miRNAs were selected as having potential relationships with oesophageal neoplasms, according to miR2Disease and dbDEMC (see Supplementary Table S2).

Kidney neoplasms are a type of cancer with an incidence increase of 43% since 1973 in the US^70^. The risk of the disorder increases with age and differs between men and women. The diagnosed number of kidney neoplasms every year has exceeded 250,000 cases^71^, among which over 80% are found to have renal-cell-carcinoma (RCC). Recent studies have found that miR-34a can be over-expressed in patients with RCC who suffer from kidney neoplasms^72^. It also showed that a combination of miR-141 with miR-155 resulted in a 97% correct classification rate, which implied reliable evidence of potential associations between miR-141/miR-155 and kidney neoplasms. To discover the potential associations between miRNAs and kidney neoplasms, we implemented DeepMDA to accomplish the prediction. The results, shown in Supplementary Table S3, were that 8 out of the top 10 and 44 out of the top 50 candidates were chosen as the kidney neoplasms related miRNAs. For example, miR-155, miR-126 and miR-20a were found over-expressed in malignant samples such as clear-cell type human renal cell carcinoma^73^. miR-145 was reported to down-regulate its target mRNA and the corresponding protein in kidney tissues^74^.

Overall, the results from LODOV and the separate case studies on three typical diseases showed satisfactory performances using DeepMDA. Unlike the traditional method, which uses prior knowledge to perform the prediction, the proposed method was capable of capturing one specific potential disease related to the relationship miRNAs without relying on any known information. Therefore, DeepMDA can be applied to a wide range of applications. To make the model more useful for the community and biologists, we also developed a web server to search for each potential disease-related miRNA that our model predicted (https://laiyifu.shinyapps.io/DeepMDA/).

## Discussions

Increasing evidence shows that miRNA genes are located at genomic regions involved in cancer, indicating that miRNAs play significant roles in the development of various diseases. Due to the limitations of previous computational models, a more effective and less costly way to predict miRNA-disease associations is required. In this study, a deep ensemble miRNA-disease association prediction (DeepMDA) framework was proposed by synthesizing heterogeneous biological networks. First, miRNA functional similarity and heterogeneous Gaussian interaction profile kernel similarities were integrated to form the miRNA similarity data and the disease semantic information. In addition, heterogeneous disease-related data were utilized to construct disease similarity data. Second, two similarity data matrices were segmented by lines separately and fed into two stacked autoencoders to learn complex high-level features. Then, the two output feature vectors from two SAEs were concatenated to form an independent feature vector, whose corresponding label was picked from a known miRNA-disease association matrix. The latter part of DeepMDA used a three-layer fully connected neural network to make the final predictions of the potential miRNA-disease associations with the feature vectors gained from two autoencoders. Both LODOCV and 5-fold cross-validation were implemented to validate DeepMDA performance. Compared with five state-of-the-art computational models and two other deep models, DeepMDA showed the best performance and good robustness compared to the other deep models. Furthermore, case studies were also implemented using several complex human diseases (colon neoplasms, oesophageal neoplasms, and kidney neoplasms), in which 47, 42 and 44 out of the top 50 predicted miRNAs, respectively, had experimentally supported evidence based on previous literature. DeepMDA can also be used to predict the miRNAs associated with isolated diseases, which could benefit human disease diagnoses and prevention.

There are several reasons that account for the reliable performance of DeepMDA. First, multiple dataset sources (more knowledge) were adopted to enlarge the miRNA and disease similarity matrices, and more data could provide more evidence when trying to predict the associations of disease-related miRNAs. Second, a deep ensemble framework was proposed to extract high-level features from traditional feature vectors and predict the potential associations using these non-linear high-level features, which improved the model’s performance compared to other state-of-the-art models.

Furthermore, the proposed model can be regarded as a more general model that may play a potential role in predicting other kinds of associations, such as lncRNA-disease, drug-targets, and so on.

## Methods

### Datasets

Biological experiments have collected many miRNA-disease associations, and multiple databases were constructed for researchers to verify the research. The human miRNA-disease dataset used in this study was downloaded from the HMDD database (June 2013)^18^. It consists of 5430 validated distinct experimental human miRNA-disease associations of approximately 495 miRNAs and 383 diseases. We used adjacency matrix *A*_*md*_ to represent miRNA-disease associations. For instance, if miRNA *m(i)* is reported to be associated with disease *d(j)* in the database HMDD, the value of *a*_*md*_ *(i,j)* is 1; otherwise, it is 0. The number of miRNAs and diseases in the database are denoted as *nm* and *nd*, respectively.

**Figure 3:**
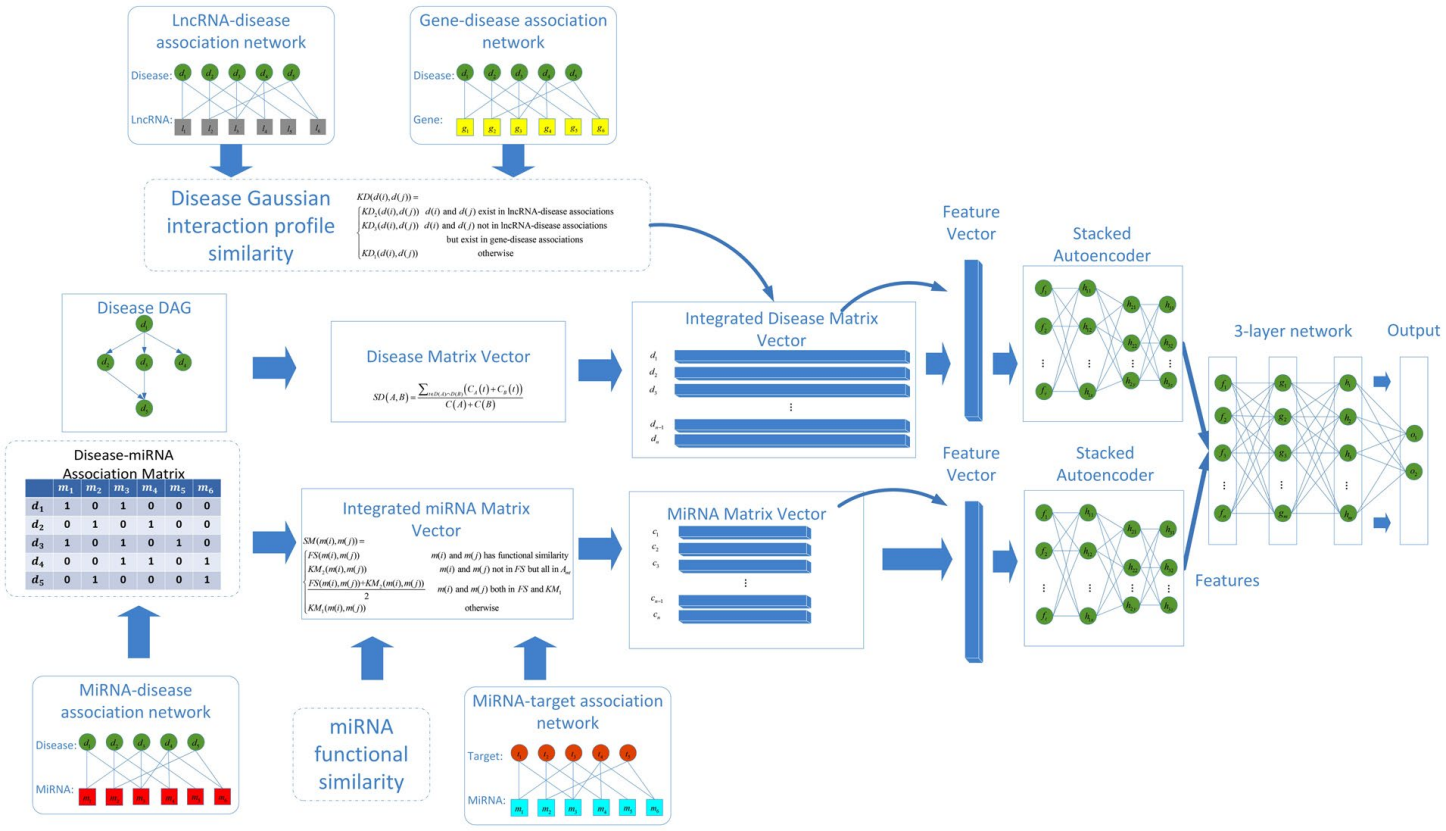
The flowchart of proposed DeepMDA. The miRNA similarity was integrated using miRNA-functional similarity and miRNA-disease association. As for disease similarity, we adopted DAG information and Gaussian interaction profile similarity information. The two input data was fed into two stacked autoencoders to learn high-level features, then merged and finally utilized a 3-layer network to infer the association between miRNAs and diseases.

We also adopted disease related long noncoding RNAs (lncRNAs) data from LncRNADisease^75^. The LncRNADisease database has integrated more than 1000 lncRNA-disease entries including 321 lncRNAs and 221 diseases from ~500 publications. Furthermore, disease-related gene data was retrieved from the DisGeNET (Version 4.0) database^76^. We chose curated gene-disease association containing 14412 genes and 10757 unique diseases from DisGeNET. Using these two data sets, we constructed two adjacency matrixes *A*_*ld*_ and *A*_*gd*_ to denote the lncRNA-disease associations and the gene-disease associations, respectively. Furthermore, due to the close relationship between miRNAs and their corresponding targets, we utilized the experimentally validated miRNA-target interaction data from miRTarBase^63^. MiRTarBase has collected more than 41000 human miRNA-target interactions, including 2649 miRNAs and 14894 targets that are validated through various studies. The adjacency matrix *A*_*mt*_ was constructed to represent miRNA-target associations. The overall design of the dataset integration is shown in Fig. 3.

### Gaussian interaction profile kernel similarity for miRNAs

Based on the assumption that similar miRNAs with similar functions tend to be associated with similar diseases, and vice versa, the interaction profile of miRNA *m(i)* is denoted by a binary vector *IP(m(i))* representing whether miRNA *m(i)* is interacted with each disease or not. Then, the kernel for the two miRNAs *m(i)* and *m(j)* are defined to calculate the Gaussian kernel similarity based on their interaction profiles, which are defined as follows:

1 $$K{M}_{1}(m(i),m(j))=\exp (-{\gamma }_{m}{\Vert IP(m(i))-IP(m(j))\Vert }^{2})$$

2 $${\gamma }_{m}=\gamma ^{\prime} /(\frac{1}{nm}\sum _{i=1}^{nm}{\Vert IP(m(i))\Vert }^{2})$$

where *γ*_*m*_ used to control the kernel bandwidth and is obtained by normalizing a new bandwidth parameter ${\gamma }_{m}^{^{\prime} }$ by the average number of associated diseases for all the miRNAs. Here, ${\gamma }_{m}^{^{\prime} }$ is set to 1 according to previous research^38^.

Likewise, similar miRNAs with similar functions tend to be related to similar target genes, and vice versa. Thus, by using miRNA-target interaction matrix *A*_*mt*_, we could also obtain 2649 miRNAs Gaussian interaction profile kernel similarity matrix. The calculation is the same as before.

3 $$K{M}_{2}(m(i),m(j))=\exp (-{\gamma }_{m}{\Vert IP(m(i))-IP(m(j))\Vert }^{2})$$

4 $${\gamma }_{m}=\gamma ^{\prime} /(\frac{1}{nm}\sum _{i=1}^{nm}{\Vert IP(m(i))\Vert }^{2})$$

where *γ*_*m*_ is calculated by normalizing ${\gamma }_{m}^{^{\prime} }$, which divided the average number of associated targets for all miRNAs. ${\gamma }_{m}^{^{\prime} }$ is set to 1 again.

### Integrate similarity for miRNAs

Because of the lack of data concerning the 495 miRNAs similarity scores, we chose to integrate miRNA functional similarity and two Gaussian interaction profile kernel similarity matrices for a new mixed similarity for each pair of miRNAs. Specifically, for a miRNA pair *m(i)* and *m(j)* that exist only in the functional similarity matrix, the miRNA functional similarity is chosen as their integrated similarity score: if *m(i)* and *m(j)* do not exist in the miRNA functional similarity matrix but both exist in the *KM*_2_ matrix, we chose their Gaussian profile kernel similarity score from *KM*_2_ as their integrated similarity score, and if *m(i)* and *m(j)* both exist in the miRNA functional similarity matrixes *FS* and *KM*_2_, the average score was calculated. If the two miRNAs do not exist in the matrixes *FS* or *KM*_2_, we adopted their Gaussian profile kernel similarity score from *KM*_1_ as the integrated similarity. The overall integrated similarity score between miRNA *m(i)* and *m(j)* is as follows:

5 $$SM(m(i),m(j))=\{\begin{array}{c}FS(m(i),m(j))\,m(i)\,and\,m(j)\,has\,functional\,similarity\,\\ K{M}_{2}(m(i),m(j))\,m(i)\,and\,m(j)\,not\,in\,FS\,but\,all\,in\,{A}_{m}\,\\ \frac{FS(m(i),m(j))\,+\,K{M}_{2}(m(i),m(j))\,}{2}\,m(i)\,and\,m(j)\,both\,in\,FS\,and\,K{M}_{1}\\ K{M}_{1}(m(i),m(j))\,\,otherwise\,\end{array}$$

### Other similarity for small dataset of miRNAs

In addition, we also obtained two miRNA similarity matrices *SM*_*F*_ and *SM*_*T*_ separately from the miRNA functional similarity matrix *FS* and the miRNA Gaussian profile similarity matrix *KM*_2_ gained from the miRNA-target associations. Specifically, 255 miRNAs in *SM*_*F*_ both appeared in HMDD and the miRNA functional similarity matrix *FS*. Similarly, there are 152 miRNAs in *SM*_*T*_ that exist in both HMDD and miRNA-target associations. These two similarity matrices *SM*_*F*_ and *SM*_*T*_ were also used to train and test our model, and their performance was evaluates using the same procedure as was used for *SM*.

### Disease semantic similarity

Many diseases’ MeSH descriptors are collected in the MeSH database, which can be downloaded from the National Library of Medicine (http://www.nlm.nih.gov)^77^. Each disease can be described as an entry item in a Directed Acyclic Graph (DAG), such as *DAG(D) = (D, T(D), E(D))*, where *T(D)* stands for the node set that includes node D itself and its ancestor nodes,*E*(*D*) represents the corresponding edge set that directly links the parent nodes to the child nodes. Here, we chose the MeSH descriptor starting with the capital letter “C” to acquire the diseases to construct the disease DAGs. Each tree number corresponds to a specific position in the DAG collected from each MeSH descriptor. In the traditional disease semantic similarity calculation^78^, disease terms in the same layer would contribute the same to the disease semantic value of disease A as an example. However, if two disease terms (disease A and B) occur in the same layer of disease DAGs but their frequency varies in all the DAGs, this causes an inaccurate measurement of the contributions of the two disease terms. Consequently, we adopted an alternative way to calculate the semantic value based on the assumption that a more frequent disease term should have a greater contribution to the semantic value of disease A, which is shown as follows:

6 $${C}_{A}(t)=-\mathrm{log}(the\,number\,of\,DAGs\,including\,t/the\,number\,of\,diseases)$$

The semantic value of disease A was calculated by summing the contribution from all the disease terms in DAG(A).

7 $$C(A)=\sum _{t\in DAG(A)}{C}_{A}(t)$$

Finally, the semantic similarity between diseases A and B can be obtained by summing the contributions of disease terms shared by the following two DAGs:

8 $$SD(A,B)=\frac{{\sum }_{t\in D(A){\cap }^{}D(B)}({C}_{A}(t)+{C}_{B}(t))}{C(A)+C(B)}$$

### Gaussian interaction profile kernel similarity for diseases

Similar to the miRNA Gaussian similarity matrix construction, the disease Gaussian interaction profile kernel similarity matrices were also computed using three association matrices, the miRNA-disease association matrix, the lncRNA-disease association matrix, and the gene-disease association matrix. Three Gaussian interaction profile kernel matrices, *KD*_1_, *KD*_2_ and *KD*_3_, were obtained and integrated to determine the overall 383 diseases in the Gaussian profile kernel similarity matrix, defined as follows:

9 $$KD(d(i),d(j))=\,\{\begin{array}{c}K{D}_{2}\,(d(i),d(j))\,d(i)\,and\,d(j)\,exist\,in\,IncRNA-disease\,associations\\ K{D}_{3}\,(d(i),d(j))\,d(i)\,and\,d(j)\,not\,in\,IncRNA-disease\,associations\,\\ \,but\,exist\,in\,gene-disease\,associations\\ K{D}_{1}(d(i),d(j))\,otherwise\,\end{array}$$

The integrated disease Gaussian interaction profile kernel similarity matrix can be used to adjust our model and improve its performance, which will be discussed in the next section.

### DeepMDA

In this study, we proposed a deep ensemble framework for miRNA-disease association prediction (DeepMDA). DeepMDA is a neural network structure composed of two parts. First, for every miRNA-disease pair *a*_*md*_ *(i, j)*, we assigned the labels of all the known miRNA-disease association pair (positive samples) to 1; otherwise, to 0. Second, the ith of the miRNA similarity matrix (i.e., the similarity data between miRNA i and all the other miRNAs) was fed into a stacked autoencoder to learn another representation, the jth row of disease similarity matrix and the jth row of integrated disease Gaussian interaction profile similarity matrix (i.e., the similarity data between disease j and all the other diseases) were concatenated into a feature vector and regarded as an independent training sample, which was fed to another stacked autoencoders. The autoencoder was a stacked deep neural network that can be trained to learn high-level biological patterns and was already implemented in some bioinformatics field such as yeast microarrays analysis^79^ and DNA Methylation state prediction^48^. Third, two separate features were then merged and integrated into a three-layer fully connected neural network to predict the label of each pair sample, which indicated whether it has a connection or not. The flowchart of DeepMDA is shown in Fig. 3.

### Stacked autoencoder

The *nm* miRNA samples correspond to *nm* rows of miRNA similarity data and the *nd* disease samples correspond to *nd* rows of the disease similarity data. These were fully connected to form a large dataset of *nm* × *nd* samples. These pair-wise samples were separately fed into two autoencoders consisting of multiple layers. Autoencoders have been widely used in capturing complex biological patterns^80^.

Assume an input data ***x*** has d dimensions. Its mapping formation is constructed as follows:

10 $${\boldsymbol{y}}=f({\boldsymbol{Wx}}+{\boldsymbol{b}})$$

where *f* is a non-linear function that maps the linear result of ***x*** to a non-linear space.

The output **y** is then projected back to form the reconstruction output **z**, which has the same shape as original input **x**. The equation is as follows:

11 $${\boldsymbol{z}}=g({\boldsymbol{W}}{\boldsymbol{^{\prime} }}y+{\boldsymbol{b}}{\boldsymbol{^{\prime} }})$$

where ${\boldsymbol{W}}{\boldsymbol{^{\prime} }}$ is the reconstruction-weighting matrix, ${\boldsymbol{b}}{\boldsymbol{^{\prime} }}$ is the reconstruction bias, and *g* is another non-linear function same as *f*. The entire reconstruction procedure needs to calculate the error; therefore, we chose the mean squared error between **x** and **z**, which can be optimized using stochastic gradient descent (SGD)^81^. All the parameters used in the network implemented greedy layer-wise learning, which learns the parameters of one layer while freezing the parameters of the other layers.

In this study we did not utilize a commonly used deep network module, such as a convolutional neural network (CNN)^47^ or a recurrent neural network (RNN)^82^ because our input was purely a similarity item corresponding to a miRNA or a disease without sequence or positional information. Therefore, CNN and RNN do not show great improvements and introduce large computational cost. As an alternative, we used fully connected layers with an activation function and dropout layers^83^ to construct an autoencoder. Dropout layers mainly help to avoid the possibility of over-fitting by randomly dropping some neuron units. The dropout rates were all set to 0.5 during model training. Finally, for a pairwise sample, we obtained two pairs of extracted high-level features using the autoencoders; these were input to the classifier to make the final predictions.

### Deep neural network

After the two autoencoders extracted two parts of high-level features, they were concatenated to form an integrated sample feature vector. Altogether, there were *nm* × *nd* samples, and the label of each sample was 1 if the miRNA-disease pair has connection according to the known relationship in the miRNA-disease association matrix otherwise 0. The combined feature vector was then fed into a feed-forward neural network consisting of three fully connected layers. We set the output dimension of each autoencoder to 64; therefore, a 128-dimensional feature vector was fed to the network. In the fully connected layer, a three-layer neural network was implemented to obtain the final prediction of the association between each miRNA and disease. The number of layers we chose here was dependent on the experiments, and the best results were obtained when the three-layer network was utilized. The predicted association possibility of each pairwise miRNA-disease sample exceeding the threshold was considered as a potential disease-related miRNA, and vice versa.

In the fully connected layer, a three-layer neuron network was implemented to get the final prediction of the association between each miRNA and disease. The predicted association possibility of each pairwise miRNA-disease sample exceeded the threshold was considered as a potential disease-related miRNA and vice versa. All the neuron units in the layer *i* was connected to the previous layer (*i* − 1) and generated outputs using non-linear transformation function *f*.

12 $${o}_{j}=f(\sum _{i=1}^{H}{w}_{i}{o}_{i}+\,{b}_{i})$$

where *H* is the number of hidden neurons, $\{{w}_{i}\,,\,{{b}_{i}\}}_{i=1}^{H}\,$are the weights and bias of neuron j which sums up all the hidden units. After one fully-connected layer, the network executed a rectified linear activation function(*ReLU*).

13 $$ReLU(x)=\{\begin{array}{c}x\,if\,x\ge 0\\ 0\,if\,x < 0\end{array}$$

Activation function ReLU is a non-linear function that can capture hidden patterns within the data^46^ and can reduce gradient vanishing in the meantime. Dropout was also used behind every fully connected layer to avoid overfitting. The final output utilized sigmoid function to make prediction of each sample, which is shown as follows:

14 $$Sigmoid(x)=\,\frac{1}{1+\,{e}^{-x}}$$

To train the model, we need to minimize the objective function in order to minimize the loss. We chose one function frequently used called cross-entropy cost function *C* ^84^.

15 $$C=-\frac{1}{n}\sum _{x}\sum _{t}[y{\rm{In}}a+(1-y){\rm{In}}(1-a)]$$

where *C* is the loss function output called cross-entropy cost function. And x is the index of the training examples and t indicates the index of different labels, y represents the true label for sample x, 0 or 1 respectively and *a* indicates the predicted output of the model for 0 or 1 label given input sample x. The more the predicted outputs approaches the true values, the less value C gets. As the cross-entropy function is non-negative, our goal is to minimize the function to get the best prediction.

Neural network models were trained using the Keras 1.0.1 library (https://github.com/fchollet/keras) with Tensorflow as the backend. The ADADELTA algorithm^85^ with a mini batch size of 200 was used to minimize the loss on the training set. The batch number was set to 200 because the model achieved the best performance using the 200-batch size. All the weights were initialized using a Gaussian distribution with a standard deviation of 0.05, and its corresponding bias was initialized ranging from unif (−1.0,0.0) as is typical. A computer with an NVIDIA Tesla K80 GPU was used to train the model. The Python code and the datasets are all available at https://github.com/sperfu/DeepMDA.

## Electronic supplementary material

Supplementary Table S1-S3
