# Supplementary material for: A deep ensemble model to predict miRNA-disease association
Source: Sci Rep. 2017 Nov 3;7:14482. doi: 10.1038/s41598-017-15235-6 (PMC5670180; doi:10.1038/s41598-017-15235-6)
Supplement: Supplementary file 1 — Supplementary Table S1-S3 [file 41598_2017_15235_MOESM1_ESM.doc]

Supplementary Information (Supplementary tables)

A deep ensemble model to predict miRNA-disease association

Laiyi Fu, Qinke Peng

**Table of Contents**

1. Supplementary Table S1
2. Supplementary Table S2
3. Supplementary Table S3

**Supplementary Table S1. We implemented DeepMDA on Colon Neoplasms for potential disease related miRNA prediction.**

| 1-25 | | 26-50 | |
| --- | --- | --- | --- |
| miRNA | Evidence | miRNA | Evidence |
| hsa-miR-125b | dbdemc | hsa-miR-181b | dbdemc;miR2Disease |
| hsa-miR-20a | dbdemc;miR2Disease | hsa-miR-124 | dbdemc |
| hsa-miR-145 | dbdemc;miR2Disease | hsa-miR-101 | dbdemc |
| hsa-miR-17 | dbdemc | hsa-miR-210 | dbdemc |
| hsa-miR-21 | dbdemc;miR2Disease | hsa-miR-141 | dbdemc;miR2Disease |
| hsa-miR-34a | dbdemc;miR2Disease | hsa-miR-222 | dbdemc |
| hsa-miR-155 | dbdemc ;miR2Disease | hsa-miR-146b | dbdemc |
| hsa-miR-146a | dbdemc | hsa-miR-199a | unconfirmed |
| hsa-miR-126 | dbdemc;miR2Disease | hsa-miR-15b | dbdemc;miR2Disease |
| hsa-miR-18a | dbdemc;miR2Disease | hsa-miR-29a | dbdemc;miR2Disease |
| hsa-miR-143 | dbdemc;miR2Disease | hsa-miR-135b | dbdemc |
| hsa-miR-31 | dbdemc;miR2Disease | hsa-miR-122 | dbdemc |
| hsa-miR-16 | dbdemc | hsa-miR-224 | dbdemc;miR2Disease |
| hsa-miR-19b | dbdemc;miR2Disease | hsa-miR-27b | dbdemc;miR2Disease |
| hsa-miR-183 | dbdemc;miR2Disease | hsa-miR-200a | dbdemc |
| hsa-miR-486 | dbdemc | hsa-miR-451a | dbdemc |
| hsa-miR-203 | dbdemc;miR2Disease | hsa-miR-106a | dbdemc;miR2Disease |
| hsa-miR-19a | dbdemc;miR2Disease | hsa-miR-133b | dbdemc;miR2Disease |
| hsa-miR-221 | dbdemc;miR2Disease | hsa-miR-193a | unconfirmed |
| hsa-miR-200b | dbdemc | hsa-miR-25 | dbdemc;miR2Disease |
| hsa-miR-223 | dbdemc;miR2Disease | hsa-miR-328 | dbdemc;miR2Disease |
| hsa-miR-92a | dbdemc | hsa-miR-34b | dbdemc;miR2Disease |
| hsa-miR-34c | miR2Disease | hsa-miR-200c | dbdemc;miR2Disease |
| hsa-miR-29b | dbdemc;miR2Disease | hsa-miR-103a | unconfirmed |
| hsa-miR-15a | dbdemc | hsa-miR-499a | unconfirmed |

**Supplementary Table S2.** **We implemented DeepMDA on Kidney Neoplasms for potential disease related miRNA prediction.**

| 1-25 | | 26-50 | |
| --- | --- | --- | --- |
| miRNA | Evidence | miRNA | Evidence |
| hsa-miR-125b | dbdemc | hsa-miR-143 | dbdemc |
| hsa-miR-21 | dbdemc;miR2Disease | hsa-miR-181a | dbdemc |
| hsa-miR-155 | dbdemc | hsa-miR-224 | dbdemc |
| hsa-miR-126 | dbdemc;miR2Disease | hsa-miR-199a | dbdemc |
| hsa-miR-34c | unconfirmed | hsa-miR-142 | unconfirmed |
| hsa-miR-145 | dbdemc | hsa-miR-17 | dbdemc;miR2Disease |
| hsa-miR-34a | dbdemc | hsa-miR-210 | dbdemc;miR2Disease |
| hsa-miR-200a | dbdemc | hsa-miR-1 | dbdemc |
| hsa-miR-29a | dbdemc;miR2Disease | hsa-miR-133b | dbdemc |
| hsa-miR-378a | unconfirmed | hsa-miR-20a | dbdemc;miR2Disease |
| hsa-miR-146a | dbdemc | hsa-miR-708 | dbdemc |
| hsa-miR-200b | dbdemc;miR2Disease | hsa-miR-138 | dbdemc |
| hsa-miR-34b | dbdemc | hsa-miR-320a | dbdemc |
| hsa-miR-29b | dbdemc;miR2Disease | hsa-miR-19b | dbdemc;miR2Disease |
| hsa-miR-16 | dbdemc | hsa-miR-125a | dbdemc |
| hsa-miR-27a | dbdemc;miR2Disease | hsa-miR-92a | dbdemc |
| hsa-miR-223 | dbdemc | hsa-miR-499a | unconfirmed |
| hsa-miR-200c | dbdemc ;miR2Disease | hsa-miR-15a | dbdemc;miR2Disease |
| hsa-miR-100 | dbdemc | hsa-miR-101 | dbdemc;miR2Disease |
| hsa-miR-29c | dbdemc;miR2Disease | hsa-miR-122 | dbdemc |
| hsa-miR-141 | dbdemc;miR2Disease | hsa-miR-99a | dbdemc |
| hsa-miR-205 | unconfirmed | hsa-miR-9 | unconfirmed |
| hsa-miR-451a | dbdemc | hsa-miR-106b | dbdemc;miR2Disease |
| hsa-miR-182 | dbdemc;miR2Disease | hsa-miR-10b | dbdemc |
| hsa-miR-203 | dbdemc | hsa-miR-133a | dbdemc |

**Supplementary Table S3. We implemented DeepMDA on Esophageal neoplasms for potential disease related miRNA prediction.**

| 1-25 | | 26-50 | |
| --- | --- | --- | --- |
| miRNA | Evidence | miRNA | Evidence |
| hsa-miR-34a | unconfirmed | hsa-miR-181b | dbdemc |
| hsa-miR-20a | dbdemc | hsa-let-7i | dbdemc |
| hsa-miR-17 | unconfirmed | hsa-miR-375 | dbdemc;miR2Disease |
| hsa-miR-18a | dbdemc | hsa-miR-15a | dbdemc |
| hsa-miR-21 | dbdemc;miR2Disease | hsa-miR-146b | dbdemc |
| hsa-miR-19a | dbdemc | hsa-let-7d | unconfirmed |
| hsa-miR-19b | dbdemc | hsa-miR-29c | dbdemc |
| hsa-miR-155 | dbdemc | hsa-miR-222 | dbdemc |
| hsa-miR-145 | dbdemc | hsa-miR-141 | unconfirmed |
| hsa-miR-92a | dbdemc | hsa-miR-7 | dbdemc |
| hsa-miR-221 | dbdemc | hsa-miR-191 | dbdemc |
| hsa-miR-126 | dbdemc | hsa-miR-101 | dbdemc |
| hsa-miR-31 | dbdemc;miR2Disease | hsa-miR-29a | dbdemc |
| hsa-miR-125b | dbdemc | hsa-miR-34b | dbdemc |
| hsa-let-7a | unconfirmed | hsa-let-7f | dbdemc |
| hsa-let-7c | dbdemc | hsa-miR-183 | dbdemc |
| hsa-miR-143 | dbdemc | hsa-miR-486 | dbdemc |
| hsa-miR-16 | dbdemc | hsa-let-7g | unconfirmed |
| hsa-miR-200b | dbdemc | hsa-miR-223 | miR2Disease |
| hsa-miR-146a | dbdemc | hsa-miR-148a | dbdemc |
| hsa-miR-29b | dbdemc | hsa-miR-203 | dbdemc;miR2Disease |
| hsa-miR-34c | dbdemc | hsa-miR-200a | dbdemc |
| hsa-miR-181a | dbdemc | hsa-miR-103a | unconfirmed |
| hsa-miR-199a | dbdemc | hsa-let-7e | unconfirmed |
| hsa-let-7b | dbdemc | hsa-miR-107 | dbdemc;miR2Disease |
